# UV-light-responsive Ag/TiO_2_/PVA nanocomposite for photocatalytic degradation of Cr, Ni, Zn, and Cu heavy metal ions

**DOI:** 10.1038/s41598-024-56059-5

**Published:** 2024-03-02

**Authors:** Mohammad Taha Dehghani, Mohammad Delnavaz

**Affiliations:** Faculty of Engineering, Civil Engineering Department, Kharazmi University, Tehran, 15719-14911 Iran

**Keywords:** Photocatalyst, Heavy metal ion, Environmental remediation, Pollution, Experimental design, Silver, Titanium dioxide, Environmental chemistry, Environmental impact

## Abstract

The rapid growth of industrialization has led to the uncontrolled pollution of the environment, and rapid action is needed. This study synthesized Ag/TiO_2_/polyvinyl alcohol (PVA) nano photocatalyst for promising light-derived photocatalytic removal of heavy metal ions. The design of experiment (DOE) was used to study the effect of important factors (pH, reaction time, and photocatalyst dosage) to maximize the final performance of the photocatalyst. In the optimized condition, the Ag/TiO_2_/PVA nano-photocatalyst removed more than 94% of Cr^6+^ in 180 min, and the efficiency was more than 70% for Cu^2+^, Zn^2+^, and Ni^2+^ metal ions. The adsorption of the heavy metal ions on the photocatalyst was described well with the Langmuir isotherm, while the pseudo-second-order linear kinetic model fitted with the experimental data. The nano-photocatalyst's stability was confirmed after maintaining its performance for five successive runs. The enhanced photocatalytic activity for the heavy metal ions removal can be attributed to the presence of metallic silver nanoparticles (electron transfer and plasmonic fields mechanisms) and PVA, which delayed the recombination of electron–hole. The synthesized ternary Ag/TiO_2_/PVA nano-photocatalyst showed promising performance for the elimination of heavy metal ions and can be used for environmental remediation purposes.

## Introduction

Environmental remediation is an unavoidable task for human beings to decrease the everyday damage of industrialization to the environment. Continuously, a large amount of pollutants (such as organic dyes, metal ions, and petroleum hydrocarbons, to name a few) are entering the surface and underground waters, soil, and atmosphere^1,2^. One of the most detrimental pollutants is the heavy metal ions (such as Hg^2+^, Pb^2+^, Cd^2+^, Co^2+^, Cr^6+^, Ni^2+^, Zn^2+^) due to their bioaccumulation ability in humans and animals bodies and the vast amount of metalizing industries around the world^3,4^. Therefore, various strategies have been adapted to date in order to detect and eliminate heavy metal ions from the environment, including sensing, chemical oxidation, chemical reduction, photocatalytic elimination, adsorption process, and many others^5–9^.

Among these heavy metal ion elimination techniques, photocatalytic strategies are low-cost, easy to endeavour, and have minimal effects on the environment^10–13^. The mechanism of photocatalytic reactions is to generate electron (e^−^) and holes (h^+^) in the band structure of the semiconductor (SC) materials and exploit these species to generate reactive species (such as oxygen and hydroxyl radicals). The generation of e^-^ and h^+^ can be done using electromagnetic radiation with energies more than the bandgap energy of the semiconductor material. TiO_2_, Si, and Ge are the first generation of semiconductor materials, which had the drawbacks of bandgap in the range of UV radiation; losing most of the available energy from the sunlight which is in the visible range^14^.

Nanostructured semiconductor materials helped to tune the bandgap energy to fabricate visible light-derived photocatalysts. In addition, nanostructured materials are able to enhance the adsorption process due to the high specific surface compared to the bulk materials. Therefore, in the last decades, numerous novel nanostructured photocatalysts have been prepared for environmental remediation applications, for instance, the photocatalysts based on g-C_3_N_4_, Bi_2_WO_6_, SnO_2_, and BiOBr, to name a few^15–17^.

Fabrication of multi-component photocatalysts is an interesting approach for enhancing photocatalytic reaction efficiency. For example, Asadzadeh-Khaneghah and Habibi-Yangjeh^18^ fabricated a g-C_3_N_4_/carbon dot-based nanocomposite with efficient performance for the degradation of various contaminants and antibiotics. In another study, a heterojunction photocatalyst based on Ag_2_MoO_4_/Bi_4_Ti_3_O_12_ nanostructures was developed capable of degrading various pollutants including various organic pollutants including methylene blue, tetrabromobisphenol A, tetracycline hydrochloride, and phenol^19^. The main mechanism for such multi-component photocatalysts based on different SC nanoparticles is to delay the recombination of electrons and holes using a designed heterojunction. The combination of semiconductor nanostructures with metallic nanoparticles has been taken into account recently to take advantage of the electron transfer from the conduction band of the SC materials to the metallic nanostructure. Another possibility is to activate the surface plasmon resonance (SPR) of plasmonic nanostructures, which can enhance the electron transitions and the performance of the whole system^20–22^. The plasmon-enhanced photocatalysis is an interesting phenomenon that has been rarely exploited in the recent years and more investigation is required.

Photocatalytic degradation has emerged as a promising approach for environmental remediation, with titanium dioxide (TiO_2_) drawing significant attention due to its exceptional properties, including chemical and thermal stability, low toxicity, and cost-effectiveness^23^. In this study, we investigate the enhancement of TiO_2_ photocatalysis through the incorporation of silver nanoparticles, aimed at reducing electron/hole pair recombination by creating a Schottky barrier at the TiO_2_ interface^24^. The integration of silver-doped TiO_2_ nanoparticles into polymers, known for their low cost, chemical inertness, mechanical strength, low density, and high durability, offers an effective means of immobilization or support. Notably, the chemical immobilization of AgTiO_2_ nanoparticles in a polyvinyl alcohol (PVA) matrix or scaffold demonstrates excellent recyclability, with minimal loss of photocatalytic activity even after multiple cycles, providing valuable insights into their potential for sustainable and efficient photocatalytic degradation^23,25,26^. This study introduces a new method by using actual industrial wastewater from a plating industry as the testing medium, showcasing the potential of the developed photocatalytic system to tackle real environmental issues. Importantly, our approach goes beyond simply testing the degradation of organic contaminants using photocatalysis, as we directly utilize real wastewater, increasing the practical significance and trustworthiness of our results. Moreover, Through the application of statistical analysis, the state with the most optimal outcome was determined.

In this study, a three-component nano-photocatalyst based on silver plasmonic nanostructure (Ag/TiO_2_/PVA) was fabricated and characterized for heavy metal removal in aqueous solutions. The synthesized nanocomposite showed promising results for the elimination of Cr^6+^, Cu^2+^, Ni^2+^, and Zn^2+^ with an efficiency of more than 90%, even for several successive runs. The synthesis condition of the Ag/TiO_2_/PVA nanocomposite was thoroughly investigated using an experimental design to yield maximum efficiency for the elimination of heavy metal ions.

## Materials and methods

### Chemicals

Silver nitrate, sodium dodecylbenzene sulfonate (SDBS), polyvinyl alcohol (PVA), and sulfochromic acid were purchased from Sigma-Aldrich. Sodium hydroxide, hydrochloric acid, acetic acid, and sulfuric acid were purchased from Merck. All of the chemicals were used in analytical grade without further purification. Distilled water (DI water) was used for the preparation of stock solutions unless mentioned.

### Synthesis of Ag/TiO_2_

First, 5 × 10^–3^ kg titanium dioxide was mixed with 1 × 10^–3^ kg SDBS in a 250 mL beaker and mixed at room temperature to make a homogenous solution. Then, the AgNO_3_ solution was prepared by dissolving 1 × 10^–4^ kg silver nitrate into 10 mL DI water, and the vial was immersed in an ice-bath container. Finally, silver nitrate solution was added dropwise to the TiO_2_ solution at 60 °C and kept for three hours. Then the beaker was transferred to the incubator at 70 °C and kept for 15 h. The obtained Ag/TiO_2_ nanopowders were transferred to a light-resistant vial and kept for further experiments.

### Synthesis of Ag/TiO_2_/PVA nanocomposite

In a typical synthesis, 5 × 10^–4^ kg of the prepared Ag/TiO_2_ powder was dispersed into 10 mL DI water followed by 5 × 10^–7^ kg PVA and kept stirring at 60 °C for 1 h. Then the solution was transferred to the incubator and kept at room temperature, and the Ag/TiO_2_/PVA nanopowders were collected after 3 h. The molar ratio of silver to TiO_2_ is 0.015, and the molar ratio of PVA to TiO_2_ is 0.001.

### Characterization

The morphological and compositional characterizations of the nanostructures were done using a scanning electron microscope (SEM, Mira 3-XMU) and X-ray diffraction (XRD, EQUINOX 3000), respectively. UV–Vis spectroscopy, diffuse reflectance spectroscopy (DRS, SHIMADZU 1800), and photoluminescence spectroscopy (PL, G9800A) were used for optical characterizations. The active surface of the final nanocomposite and its functional groups were analyzed using Brunauer–Emmett–Teller (BET, JW-BK132F) and Fourier transform infrared (FT-IR, EQUINOX 55), respectively. The initial concentration of contaminants in actual wastewater samples is quantified using Inductively Coupled Plasma Optical Emission Spectroscopy (ICP-OES, VISTA-MPX).

### Photocatalytic activity

The photocatalytic activity of the nanostructures for the removal of heavy metal ions (Cr^6+^, Cu^2+^, Ni^2+^, and Zn^2+^) was evaluated in a custom-built chamber for irradiation of ultraviolet energy using a UV-C lamp (253 nm, 8 W, 150 mA). In a typical experiment, 100 mL of wastewater from the plating industry in Yazd Province (Iran) was mixed with 0.1 g Ag/TiO_2_/PVA nanocomposite for three hr in the built setup under UV irradiation. Then, the supernatant of the samples was collected after centrifuge and analyzed (ICP-OES, VISTA-MPX) to measure the removal percentage ($\eta$) of the metal ions [Eq. (1)].

1 $$\eta =\frac{\left({C}_{o}- {C}_{t}\right)}{{C}_{o}}\times 100$$

where C_0_ and C_t_ is the initial and final concentration of metal ions before and after photocatalytic reaction, respectively. The initial concentration of the wastewater for Cr^6+^, Cu^2+^, Ni^2+^, and Zn^2+^ ions were 141 ppm, 33 ppm, 19 ppm, and 66 ppm, respectively. In addition, the adsorption isotherms and removal kinetics of the heavy metal ions in the presence of Ag/TiO_2_/PVA nanocomposite have been studied in detail.

### Experimental design

For further elucidation of the photocatalytic removal of heavy metal ions from plating wastewater, the effect of pertinent factors (pH, photocatalyst concentration, and irradiation time) was studied using response surface methodology (RSM, Design-Expert software). Central composite design (CCD) with central, factorial, and axial points was used to design the model by considering five different levels for each factor. The range of parameters in the model can be seen in Table 1, and twenty runs were designed using the Design-Expert software (see Supplementary Information—Table S1).

**Table 1:** The range of parameters in the designed model.

Parameters	Dimension	Code	Levels				
			+ α	+ 1	0	− 1	− α
Irradiation time	min	A	360	300	210	120	60
pH	–	B	8	7	5.5	4	3
Nanocatalyst concentration	g/L	C	0.2	0.16	0.11	0.05	0.02

All of the designed runs were done in triplicates, and the removal percentage ($\eta$) of the metal ions was reported as the mean value for each run as the response. Then, a second-order polynomial regression model was used to describe the effect of factors on the response as Eq. (2):

2 $$ Y = \beta_{0} + \sum\nolimits_{i = 1}^{k} {\beta_{i} x_{i} } + \sum\nolimits_{i = 1}^{k} {\beta_{ii} x_{i}^{2} } + \sum\nolimits_{i = 1}^{k - 1} {\sum\nolimits_{j = 2}^{k} {\beta_{ij} x_{i} } x_{j} + \,} \varepsilon $$

In this equation, *x*_*i*_*, x*_*i*_^*2*^*, x*_*i*_*x*_*j*_*,* and *β*_*0*_*, β*_*i*_*, β*_*ii*_*,* and *β*_*ij*_ are the independent variables and intercept terms, respectively. The analysis of variance (ANOVA) was used to evaluate the significance and consistency of the predicted model (P-value < 0.05 and F-value > 0.05)^27^.

## Results and discussions

### Characterization of the synthesized Ag/TiO_2_/PVA nanocomposite

**Figure 1:**
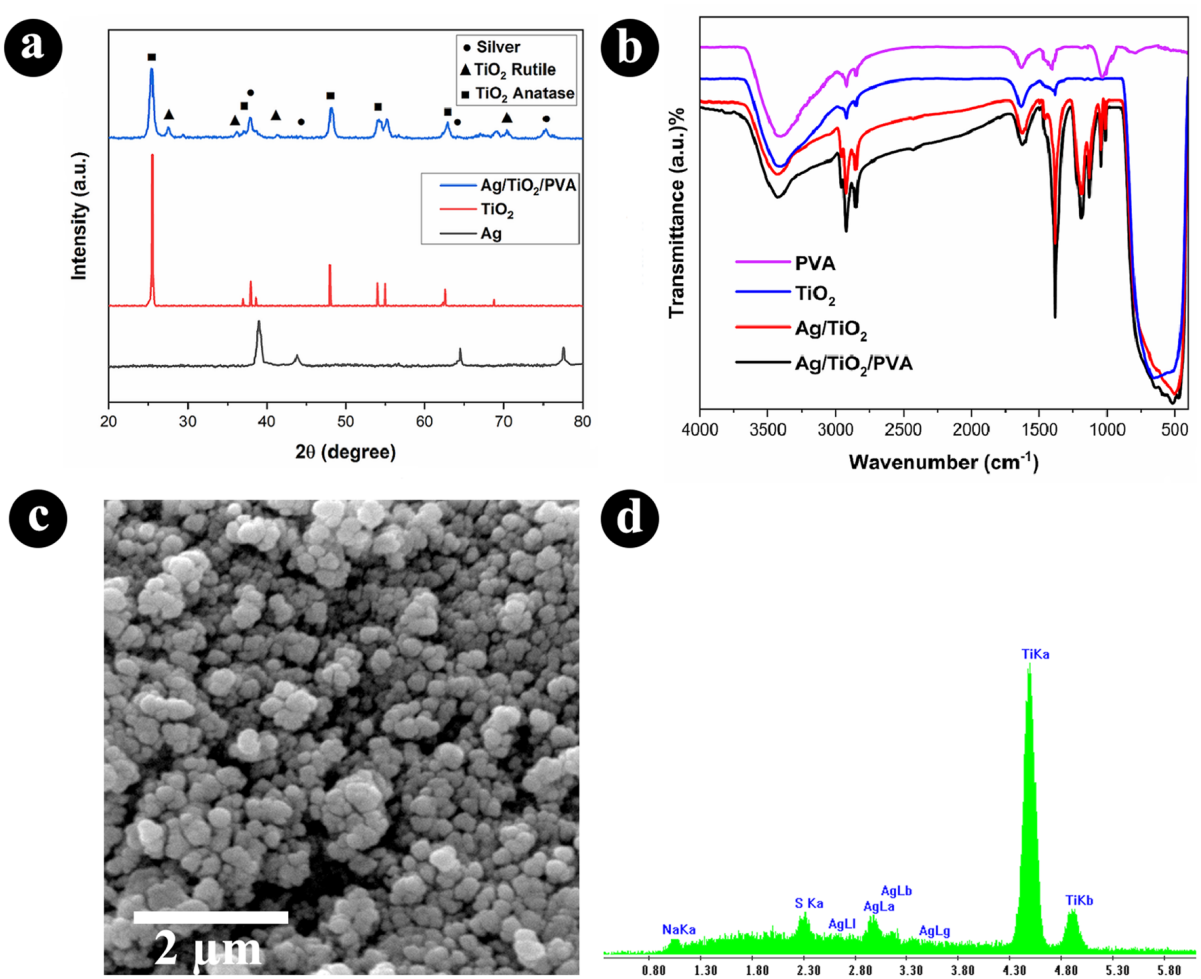
X-ray diffraction of the Ag, TiO_2_ and synthesized Ag/TiO_2_/PVA nanocomposite (**a**) and FT-IR spectra of the TiO_2_, Ag/TiO_2_, and Ag/TiO_2_/PVA (**b**). Panel (**c**) and (**d**) show the SEM and EDS spectra of the Ag/TiO_2_/PVA nanocomposite.

The composition of the synthesized Ag/TiO_2_/PVA nanocomposite was characterized using X-ray diffractometry. According to the Fig. 1a, the standard peaks of Ag at 2θ of 38.1, 44.2, 64.7, and 77.4 degrees were observed, which can be attributed to the (111), (200), (220), and (311) planes of face-centered cubic silver^28^. The anatase seems to be the dominant phase of titanium dioxide according to the intensity observed at 25.4, 48.0, 54, 62.9, and 75.1 degrees for (101), (200), (211), (118), and (220) planes of tetragonal phase^29^. The diffraction of (110), (211), and (301) planes of the rutile phase were observed and assigned in Fig. 1a. The analysis confirmed the successful synthesis of Ag/TiO_2_ nanostructure, and the FT-IR analysis was used to confirm the presence of PVA due to its amorphous structure. According to the Fig. 1b, symmetric and asymmetric stretching vibration and bending vibration of –OH can be seen at 3485 cm^−1^ and 1690 cm^−1^, respectively. The broad band at 480 cm^−1^ can be attributed to the Ti–O–Ti bond, confirming the formation of TiO_2_ nanoparticles. The spectrum of Ag/TiO_2_ nanostructure, shows new bands emerged at 2925 cm^−1^, 2853 cm^−1^, 1383 cm^−1^, and 1045 cm^−1^ attributed to the C–H and C–O groups of the used surfactant (SDBS). The presence of PVA in the final nanocomposite was confirmed from the observed enhancement in the intensity of bands at 2925 cm^−1^, 2853 cm^−1^, 1383 cm^−1^, and 1045 cm^−1^, which can be attributed to the C–H groups of aliphatic compounds in PVA. From the scattering electron microscopy (SEM) analysis (Fig. 1c), The average size of Ag/TiO_2_/PVA nanoparticles synthesized in this study has been determined to be approximately 200 nm, based on a thorough analysis of several SEM images obtained from different regions of the nanoparticles. The SEM images were captured at high magnification, allowing for precise measurements of individual nanoparticles using advanced image analysis techniques. The presence of PVA in the nanocomposite system may have influenced the size distribution by providing stabilization or dispersion effects. However, further research is required to investigate the specific mechanisms behind this phenomenon.

it can be inferred that after the synthesis of the final nanocomposite, the size of Ag/TiO_2_ nanoparticles were increased from the average size of 150 nm to 200 nm, which can be attributed to the presence of PVA on the Ag/TiO_2_/PVA nanocomposite. In addition, by elemental analysis of surfaces in SEM that is performed using energy dispersive spectroscopy (EDS), which measures the energy and intensity distribution of X-ray signals generated by the electron beam striking the surface of the specimen, the EDS analysis confirmed the elemental Ag and Ti in the final nanocomposite (Fig. 1d). The SEM images showed the semi-spherical morphology of the particles with observable porosity. To shed more light on the structure of the nanostructures, BET analysis was used (see the SI—Table S2), and a specific surface of 23.29 m^2^/gr was measured.

**Figure 2:**
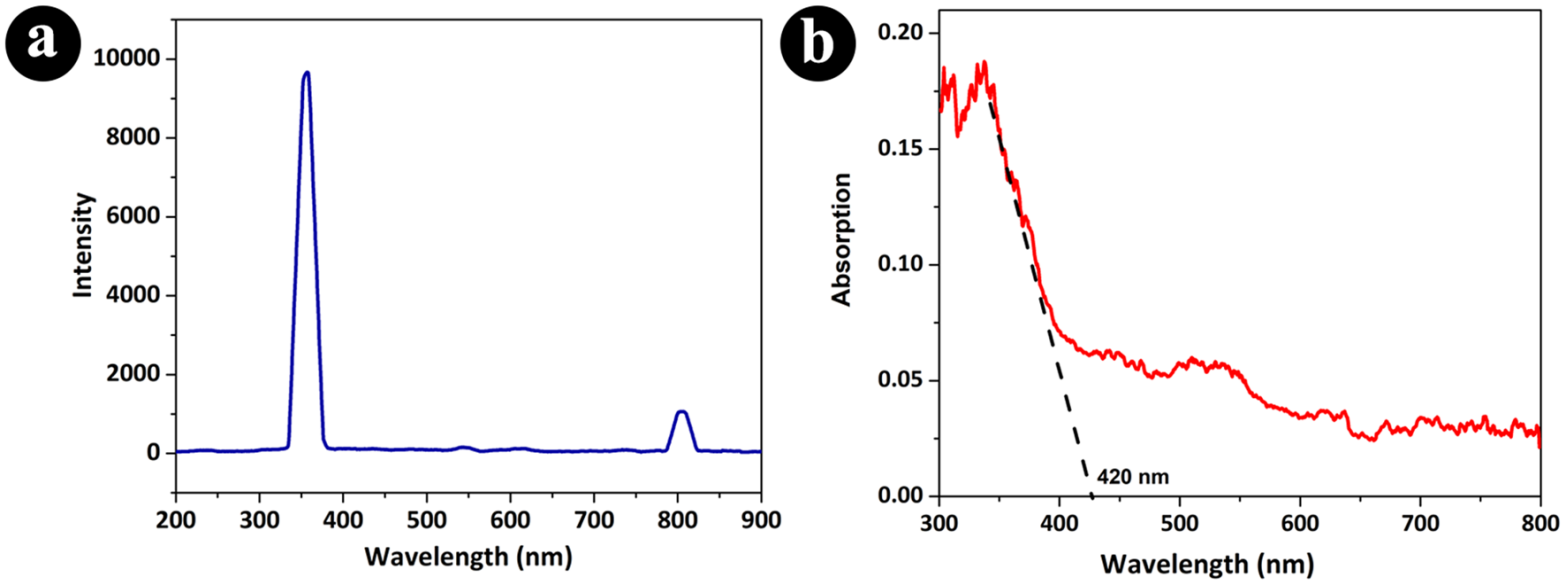
PL (**a**) and DRS (**b**) spectra of the synthesized Ag/TiO_2_/PVA nanocomposite.

The optical characteristics of the final nanocomposite were analyzed using photoluminescence (PL) spectroscopy and diffusive reflectance spectroscopy (DRS) (Fig. 2). The PL spectrum shows two emissions at 352 nm and 800 nm. The PL emission spectra have been widely used to investigate the efficiency of charge carrier trapping, immigration and transfer, and to understand the fate of electron/hole pairs in semiconductor particles^30^. Based on similar research, The observed red shift in the PL peak from 342 nm for pure TiO_2_ to 352 nm in the Ag/TiO_2_/PVA composite indicates a change in the electronic structure and optical properties of the material^31^. This shift is often associated with the modification of the bandgap and the creation of new energy levels within the material due to the presence of silver and PVA. The introduction of silver and PVA may lead to the formation of defects, impurity levels, or surface states within the TiO_2_ matrix, which can affect the recombination processes of charge carriers and subsequently alter the emission characteristics. Additionally, the presence of silver nanoparticles, in combination with the PVA matrix can also contribute to the observed PL peak shift through plasmonic effects^32,33^.

On the other hand, the peak at 800 nm in the PL spectrum is associated with the photoluminescence behavior of the TiO_2_ component within the composite. This peak demonstrates that titanium dioxide maintains its characteristic photoluminescence properties within the composite, despite the presence of silver and polyvinyl alcohol. The existence of this peak highlights the individual contribution of TiO_2_ to the composite's overall photoluminescence spectrum, providing valuable insight into its role in shaping the nanocomposite's optical behavior^34^. Understanding the mechanisms behind this red shift is crucial for elucidating the interactions between the components in the composite and their impact on the material's optical behavior.

Further analysis and characterization, including diffuse reflectance spectroscopy (DRS) as previously mentioned, can provide a more comprehensive understanding of the electronic transitions and band structure modifications in the Ag/TiO_2_/PVA composite. The DRS analysis revealing a bandgap energy of approximately 2.9 eV for the Ag/TiO_2_/PVA nanocomposite provides crucial information about the modification of the material's electronic structure. This lower bandgap energy compared to pristine TiO_2_ (3.5 eV) suggests that the addition of silver and polyvinyl alcohol has influenced the band structure of the composite^35^. The reduction in bandgap energy can be attributed to various factors, such as the formation of defect levels, changes in the electronic configuration, or the influence of surface plasmon resonance induced by the silver nanoparticles^20^.

These modifications in the band structure are consistent with the observed red shift in the photoluminescence peak, as they indicate alterations in the energy levels and electronic transitions within the material. Absolutely, the decrease in bandgap energy of the Ag/TiO_2_/PVA nanocomposite has significant implications for its photocatalytic activity, particularly in the visible light range. A lower bandgap energy allows the material to absorb a broader spectrum of light, including visible light, which is essential for enhancing its photocatalytic performance under natural sunlight or indoor lighting conditions. The delay in electron–hole recombination, facilitated by the modified band structure and the presence of PVA and silver atoms, can lead to improved efficiency in the generation and utilization of photoinduced charge carriers. This, in turn, enhances the photocatalytic activity of the nanocomposite, making it more effective in processes such as pollutant degradation, water purification, and other environmental remediation applications. The ability of the Ag/TiO_2_/PVA nanocomposite to harness visible light for photocatalysis, coupled with the reduced electron–hole recombination, underscores its potential for practical applications in sustainable energy and environmental technologies^36–38^.

### Preliminary study on the photocatalytic activity of Ag/TiO_2_/PVA nanocomposite for the removal of heavy metal ions

**Figure 3:**
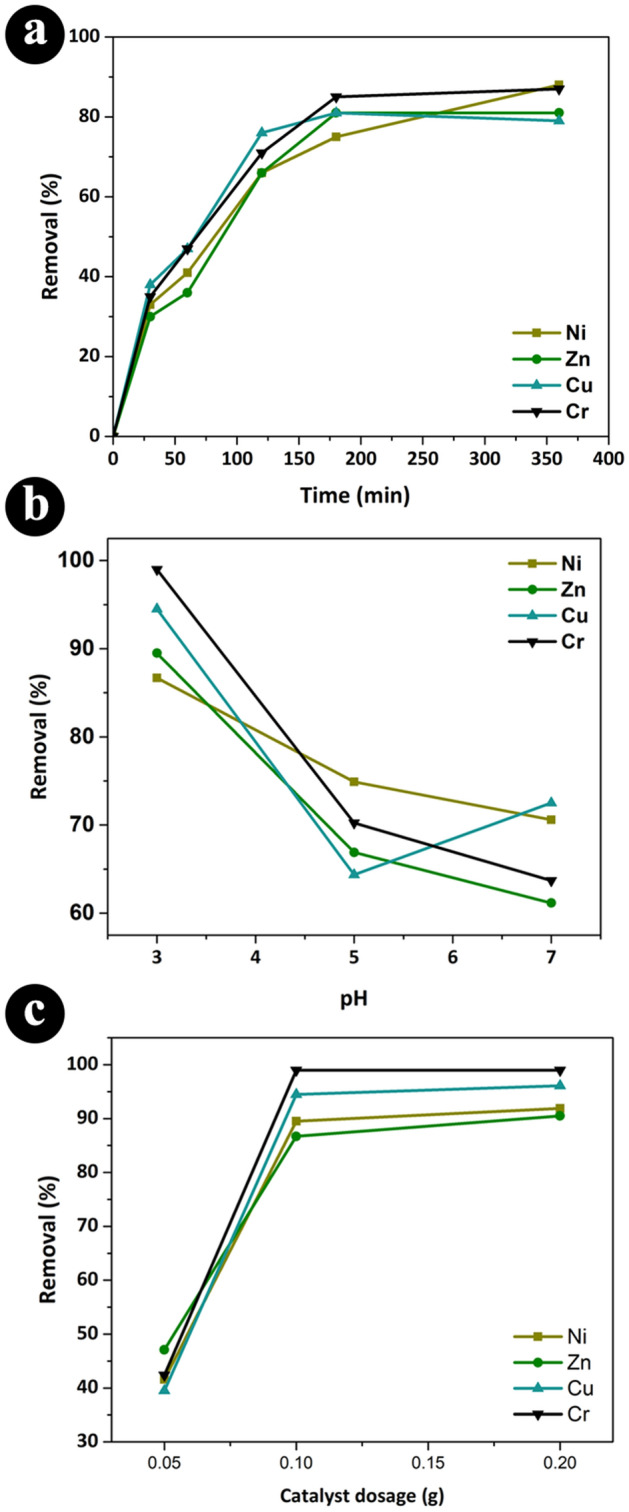
The effect of reaction time, pH, and photocatalyst dosage on the removal percentage of heavy metal ions.

The synthesized photocatalyst was used for the removal of heavy metal ions from industrial wastewater containing Cr^6+^, Cu^2+^, Ni^2+^, and Zn^2+^ metal ions. The effect of pertinent factors, including lamp type, removal duration, pH, and photocatalytic dosage, was studied. As it can be seen in Fig. 3a, more than 70% of the heavy metal ions were removed after 180 min upon irradiation by a UV lamp in the presence of 0.1 g Ag/TiO_2_ nanocomposite. The pH of the solutions seems to play a crucial role in the photocatalytic activity of the nanostructure as lower pH values were more ideal for heavy metals removal (see Fig. 3b). This is consistent with previous studies on the effect of pH on the elimination of metal ions, as the metallic ions are more stable in an acidic medium^39^. Increasing the amount of photocatalyst to more than 0.1 g seems not important, as confirmed from the obtained results in Fig. 3c. UV-C lamp showed superior performance in comparison with UV-A and sunlight, as it can be seen in Table 2.

**Table 2:** Heavy metal ions removal efficiency in the presence of UV-C, UV-A, and sunlight irradiation.

	UV-C	Sunlight	UV-A
Cr	99	60.2	49
Cu	94.5	63.7	47
Ni	86.7	46.4	35
Zn	89.5	53.0	37

Reaction time, pH, and photocatalysts dosage was set at 3 h, 3, and 0.1 g, respectively.

Based on the obtained preliminary results, the time of the photocatalytic reaction, pH, and photocatalyst dosage seems to be important factors for enhancing the photocatalytic elimination of heavy metal ions. In this regard, more systematic runs were designed by DOE to capture the possible interaction between these parameters.

### Design of experiment

As mentioned earlier, twenty different runs were suggested from the experimental design based on the parameters' range written in Table 1. The results for the elimination of each metal ion after conducting the experiment can be seen in Table S3, where the significance of the model for each ion (Cr^6+^, Cu^2+^, Ni^2+^, and Zn^2+^) was assessed from the ANOVA, and p-values (lower than 5%). In the case where the developed model was significant, surface plots were used to give more information on the interaction between the parameters.

The obtained ANOVA table for each metal ion can be seen in the supplementary information (Tables S4–S7). As it can be seen, in the case of Cr^6+^ ions, all of the linear and quadratic terms were significant (p-value < 0.05), and the insignificant F-value (F-value > 0.05) proved the adequacy of the model. Therefore, the written polynomial in Eq. (3) can describe the effect of factors on the response (elimination of Cr^6+^ ions) with a regression value of 97.23%:

3 $$ \left( {{\text{Cr}}^{6 + } {\text{removal}}} \right) = { 1}.{577 } + \, 0.00{\text{4A }} + \, 0.00{\text{6B }} + \, 0.00{\text{5C }} - \, 0.000{\text{3AB }} + \, 0.00{\text{1AC }} - \, 0.00{\text{1BC }} - \, 0.00{\text{7A}}^{{2}} - \, 0.0{\text{14B}}^{{2}} - \, 0.00{\text{2C}}^{{2}} $$

**Figure 4:**
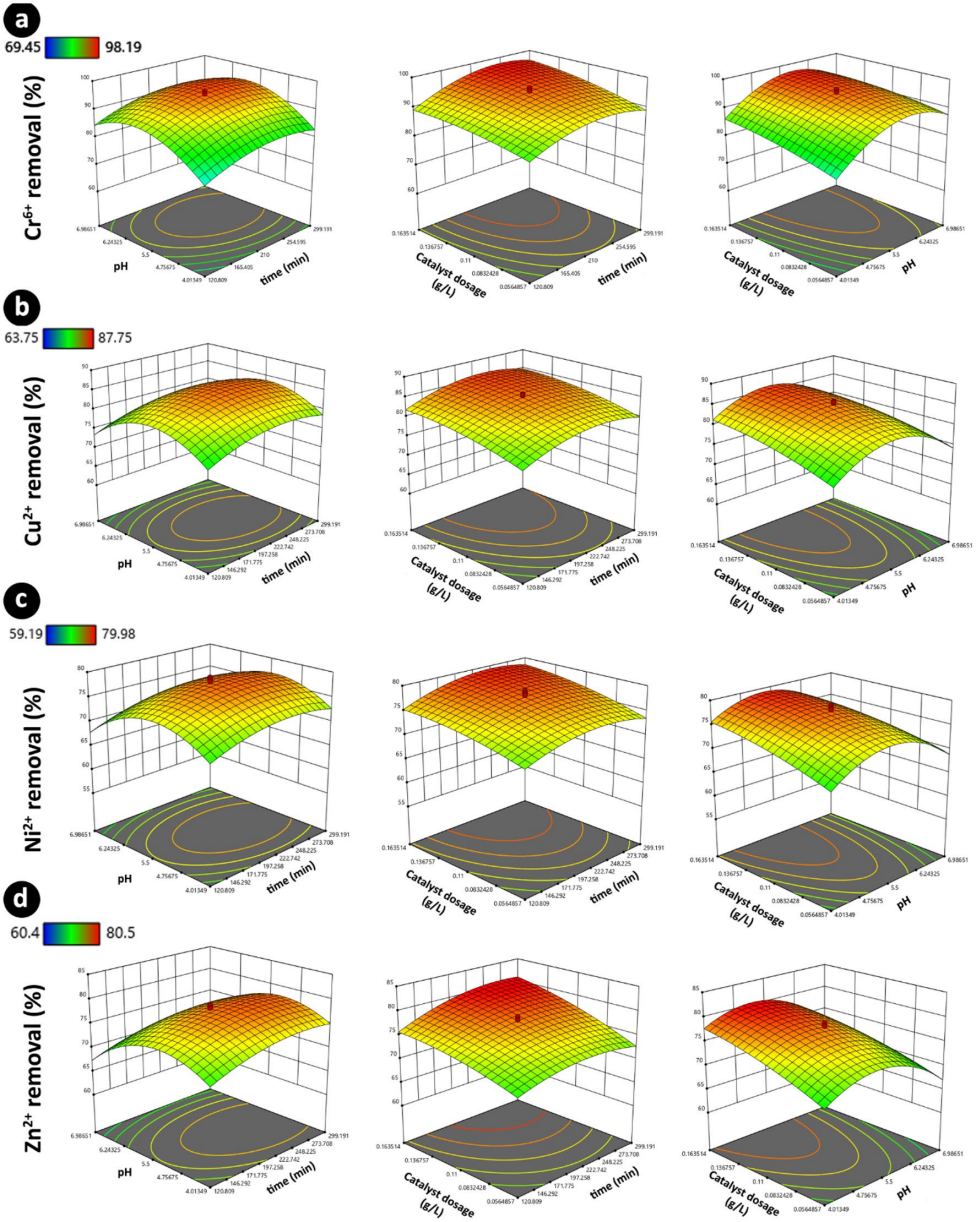
Surface plots for the removal of heavy metal ions based on the interaction of the reaction time, pH, and catalyst concentration. The legend bar for each row shows the corresponding color for the minimum and maximum on each plot.

In the above-mentioned equation, the coefficient of each term can be used to justify its level of importance on the model. For instance, in the case of Cr^6+^ ion, pH seems to be the most important factor, followed by catalysis dosage and irradiation time. The surface plot (Fig. 4a) can be used to study the interactive effect of parameters on each other graphically. For instance, it can be inferred that for efficient removal of Cr^6+^ ions, pH in the range 5–6 should be used regardless of the value of other parameters.

In the case of Cu^2+^ ions, again, all of the linear and quadratic terms were significant, and the regression value of 91% was obtained for the model Eq. (4) can be used to describe the model:

4 $$ \left( {{\text{Cu}}^{2 + } {\text{removal}}} \right) = { 84}.{84}0 \, + { 1}.{\text{499A }} - { 1}.0{\text{52B }} + { 2}.{4}0{\text{2C }} - \, 0.{\text{213AB }} - \, 0.0{\text{26AC }} - \, 0.{\text{511BC }} - { 2}.{82}0{\text{A}}^{{2}} - { 6}.{41}0{\text{B}}^{{2}} - { 1}.{\text{179C}}^{{2}} $$

As can be seen, catalyst dosage, irradiation time, and pH are the three most important factors in the case of Cu^2+^ ions elimination. The surface plots (Fig. 4b) show the catalyst dosage should be in the optimum range (> 0.1 g/L) to achieve efficient removal of Cu^2+^ ions. Figure 4b shows that if the pH value is lower than 5 or higher 6, the final Cu^2+^ removal is lower than 70%.

In the case of Ni^2+^ ions, it was inferred that if the pH was outside of the optimal range (5–6), low removal efficiency would be obtained, regardless of the level of other parameters (see surface plots in Fig. 4c). The polynomial for describing the effect of factors on Ni^2+^ removal was obtained as follows:

5 $$ \left( {{\text{Ni}}^{2 + } {\text{removal}}} \right) = { 77}.{732 } + { 1}.{\text{293A }} - { 1}.{\text{155B }} + { 2}.{\text{171C }} + \, 0.0{1}0{\text{AB }} + \, 0.{\text{337AC }} - \, 0.{\text{687BC }} - {2}.{\text{164A}}^{{2}} - { 5}.{3}0{\text{7B}}^{{2}} - \, 0.{\text{868C}}^{{2}} $$

With a close observation in Fig. 4d, it can be inferred that in a short reaction time and catalyst dosage, it is impossible to obtain proper Zn^2+^ removal and the final efficiency is below 70%.

For Zn^2+^ removal, according to the ANOVA table, the interaction term between irradiation time and catalyst dosage was significant. This is consistent with the obtained results in Fig. 4d, where it can be inferred that at a lower catalyst dosage, a longer reaction time should be used to obtain efficient Zn^2+^ removal. The proposed polynomial (regression value of 98%) for this ion can be written as:

6 $$ ({\text{Zn}}^{2 + } {\text{removal}}) \, = { 77}.{99}0 \, + { 1}.{\text{683A }} - { 2}.{\text{148B }} + { 3}.{\text{173C }} - \, 0.{\text{238AB }} + \, 0.{\text{923AC }} - 0.{\text{486BC }} - { 1}.{89}0{\text{A}}^{{2}} - { 5}.{\text{148B}}^{{2}} - 0.{\text{971C}}^{{2}} $$

From the above equation, it can be inferred that pH is one of the most important parameters for Zn^2+^ removal, which is consistent with the surface plots (Fig. 4d) that suggest an ideal range of pH (5–6) is required for the efficient ion removal.

Overall, based on the obtained results, it was inferred that the final response (removal efficiency) experienced a wide range of behavior from the factors. The optimization process from the software was implemented to propose the appropriate level of parameters for obtaining maximum efficiency for all of the studied heavy metal ions. Based on the software optimization, the optimal values of the parameters were obtained as written in Table 3. The experiments were conducted in triplicate in the proposed level of factors, and the efficiency values of 94%, 85%, 77%, and 77% were obtained for, Cr^6+^, Cu^2+^, Zn^2+^, and Ni^2+^, respectively.

**Table 3:** The efficiency of heavy metal ions removal in the optimal condition for three distinct runs.

Run	Time (min)	pH	Catalyst dose (g/L)	Cr removal (%)	Cu removal (%)	Zn removal (%)	Ni removal (%)
1	236	5.4	0.09	94.24	85.01	77.08	76.92
2	236	5.4	0.09	94.8	84.09	77.54	77.06
3	236	5.4	0.09	93.25	83.75	76.82	76.18

### The comparison of photocatalytic activities for Ag/TiO_2_/PVA in this work with the previously reported photocatalysts in other works

Examples of photocatalysts such as TiO_2_, Ag, and PVA used for the photocatalytic degradation of various pollutants are summarized in Table 4.

**Table 4:** The comparison of photocatalytic activities for Ag/TiO_2_/PVA in this work with the previously reported photocatalysts in other works.

Type of catalyst	Target metals	Condition	Removal	Reference
TiO_2_	Zn(II), Cd(II)	Hydrolysis particles with size of 10–50 nm, Surface area 208 m^2^/g	Capacity: 15.3 (Zn) and 7.9 (Cd) mg/g	^40^
Ag/TiO_2_	Cd(II), Ni(II), Zn(II), Mn(II) and Cu(II)	UV light, 120 min	100, 96, 65.13, 58.22 and 56.20%	^41^
TiO_2_/PVA	Methylene Blue, Cu(II)	Visible light, 60 min	96.4, 99% Or 96, 1620 mg/g	^42^
TiO_2_/PVA	Methylene Blue	pH 8, 8 min	97.1%	^43^
Ag-PVA/TiO_2_	Cd(II)	A composite film	Rang of 0–1 μM	^44^
Ag/TiO_2_/PVA	Cr(VI), Cu(II), Zn(II), Ni(II)	UV-C light, pH 5.4, 236 min	94%, 85%, 77%, 77% respectively	This research

### Kinetics study

#### Adsorption isotherms

**Figure 5:**
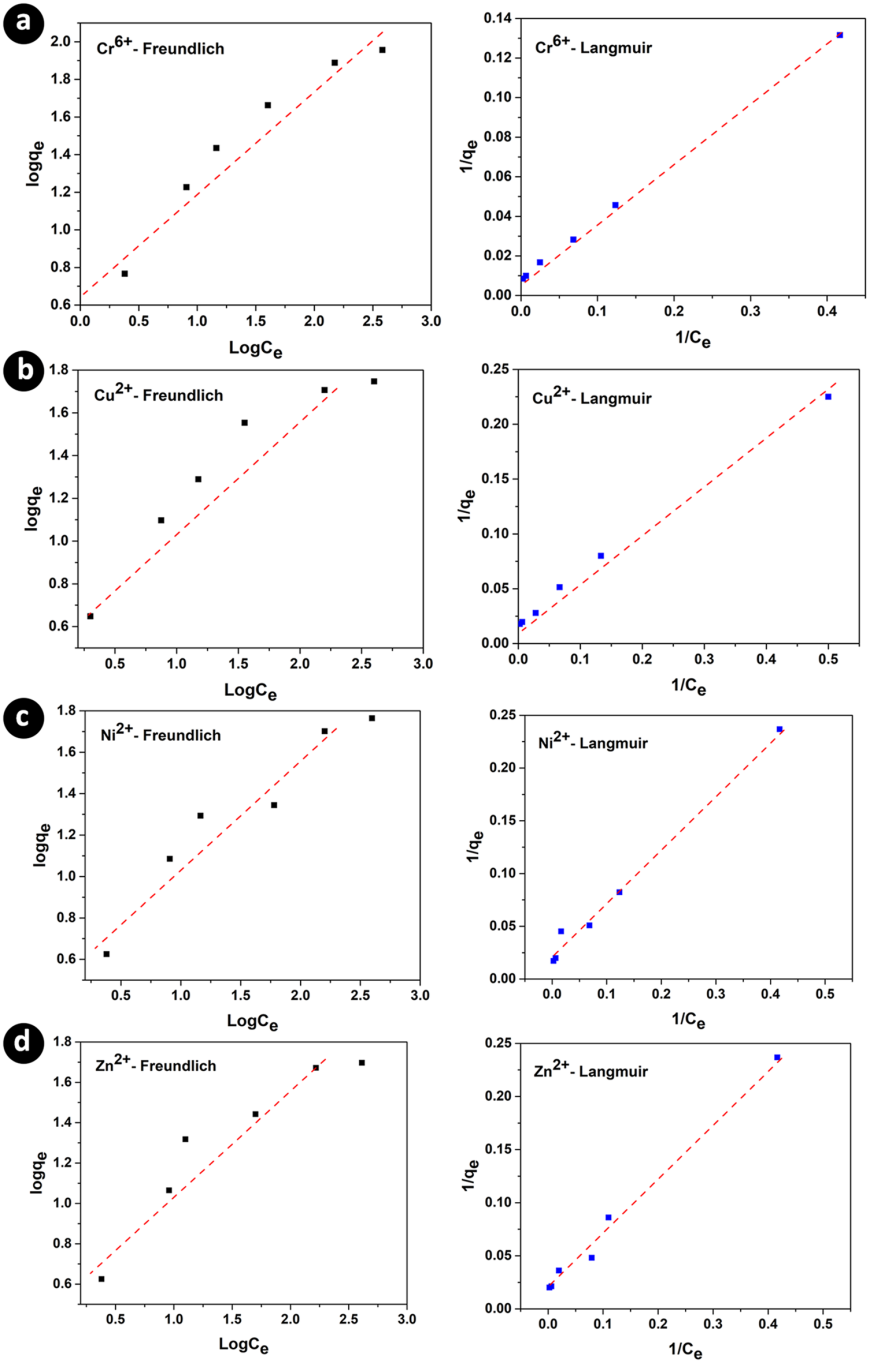
The Fitness of the linear Langmuir and Freundlich equations with the experimental data for the removal of heavy metal ions. Panels (**a**, **b**, **c**, and **d**) correspond to the data for the Cr^6+^, Cu^2+^, Ni^2+^, and Zn^2+^ ions, respectively.

After the optimization of the pertinent factors for the removal of heavy metal ions, the kinetics of the reaction was investigated to shed more light on the photocatalytic reactions. The experimental data were fitted with the linear forms of Langmuir and Freundlich isotherms [Eqs. (7) and (8)], and according to the obtained data in Fig. 5 and Table 5, the Langmuir isotherm fits well (regression values) with the experimental data. In this regard, it can be concluded that the adsorption of metal ions on the surface of Ag/TiO_2_/PVA nanocomposite is monolayer and homogenous.

7 $$ \frac{1}{{\mathop q\nolimits_{e} }} = \frac{1}{{\mathop q\nolimits_{\max } }} + \frac{1}{{\mathop q\nolimits_{\max } \mathop K\nolimits_{L} \mathop C\nolimits_{e} }} $$

8 $$ \ln \left( {\mathop q\nolimits_{e} } \right) = \ln \left( {\mathop K\nolimits_{F} } \right) + \frac{1}{{\mathop n\nolimits_{F} }}\ln \left( {\mathop C\nolimits_{e} } \right) $$

**Table 5:** The fitted parameters for Langmuir and Freundlich isotherms.

	Freundlich isotherm			Langmuir isotherm		
	q_max_ (mg g^−1^)	K_L_ (L g^−1^)	R^2^	1/n	K_F_ (mg g^−1^)	R^2^
Cr	119.332	0.028	0.999	0.530	5.135	0.925
Ni	47.939	0.040	0.988	0.483	3.756	0.909
Cu	43.936	0.056	0.966	0.473	4.508	0.893
Zn	1.046	0.037	0.989	0.460	4.117	0.874

In these equations, C_e_ and q_e_ are the equilibrium concentration of metal ions (mg/L) and the adsorption capacity of the adsorbent (mg/g). While q_max_, K_L_, K_F_, and η_F_ are the maximum adsorption capacity (mg/g), Langmuir's equilibrium constant, and Freundlich equilibrium constants, respectively.

The adsorption rate was investigated by fitting the pseudo-first-order and pseudo-second-order linear kinetic models. Based on the obtained results, the pseudo-second-order linear kinetic model fitted more (regression values more than 95%) with the experimental data (Table S8 and Fig. 5).

### The recyclability of the synthesized Ag/TiO_2_/PVA nano-photocatalyst

**Figure 6:**
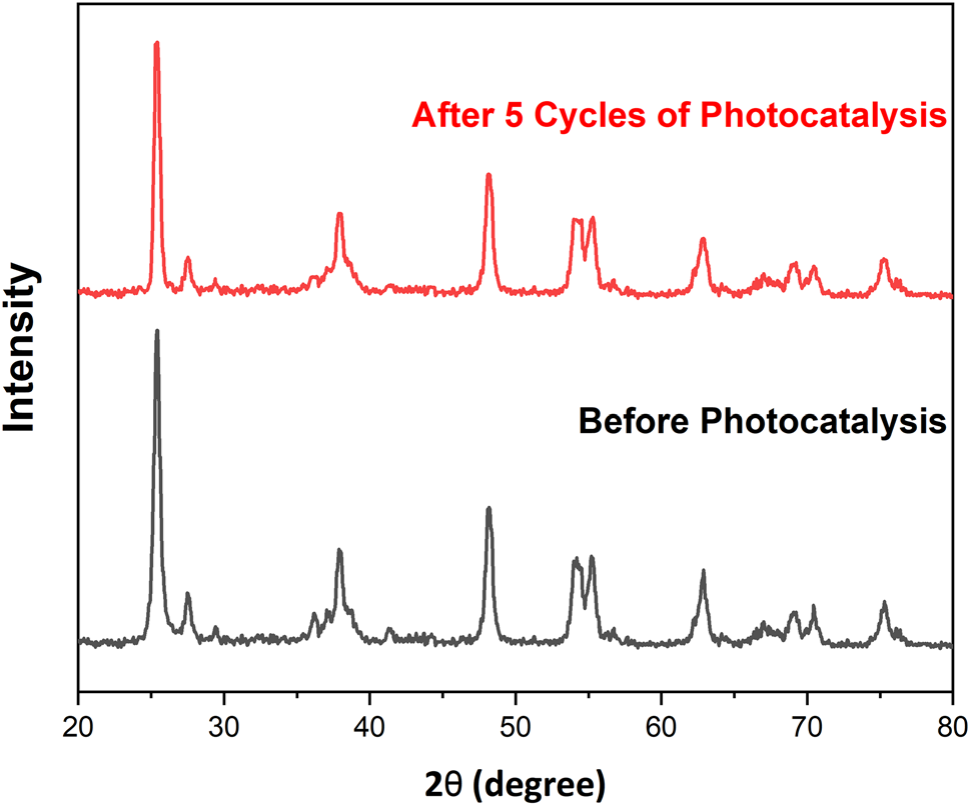
XRD patterns of Ag/TiO_2_/PVA before and after 5 cycles photocatalytic reactions.

The possibility to reuse the photocatalyst is a crucial property for its practical application. To assess this ability for the synthesized photocatalyst, the Ag/TiO_2_/PVA nanocomposite was used for the elimination of Cu^2+^ ions in the optimal condition for five consecutive runs. The system washing procedure involved collecting the sediments that settled after centrifugation and subsequently dissolving them in a solution containing diluted sulfochromic acid following each test period. The pH of the solution was then adjusted to a neutral state to prevent damage to the filter paper. Upon passing the neutral solution through filter paper, residue was observed. The remaining material on the strainer was then dried in an oven at 70 degrees for 3 h and reused. Based on the obtained results, the performance of the photocatalyst was decreased by only 5% after five consecutive runs (see the SI—Fig. S1), which proved the stability and potential application of the nano-photocatalyst for heavy metal ions removal (Fig. 6).

### Mechanism of Ag/TiO_2_/PVA nano-photocatalyst for heavy metal ions removal

**Figure 7:**
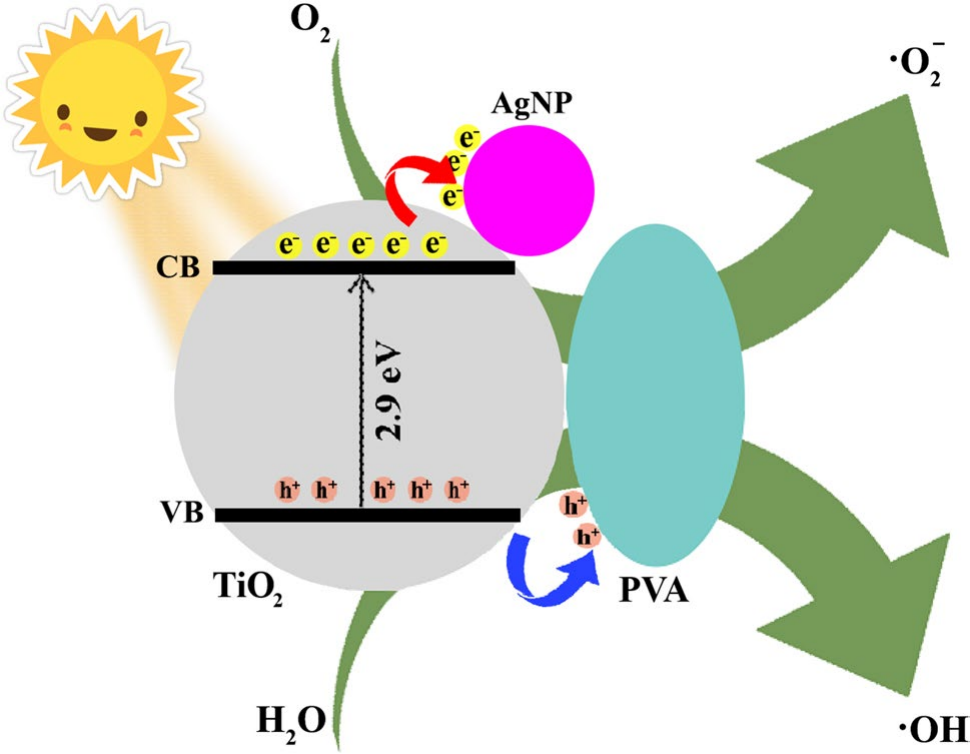
Schematic representation of the mechanism proposed for the generation of e^-^-h^+^ and the reactive oxygen species using the Ag/TiO_2_/PVA nanocomposite.

The schematic of the photocatalytic reduction of heavy metal ions using nanostructured Ag/TiO_2_/PVA is shown in Fig. 7. At first, the oxygen, hydroxyl, and heavy metal ion adsorbed on the nanocomposite surface. It is worth mentioning that even in the dark condition, the nanocomposite showed the ability to remove the heavy metal ions (mainly lower than 30%), which is due to the adsorption of species on the surface of the Ag/TiO_2_/PVA nanocomposite. Upon the irradiation of the nano-photocatalyst, the valence electrons in TiO_2_ can move up to the conduction band with higher energies. As soon as the electron transition occurs, the generated electrons (e^−^) and holes (h^+^) are looking to recombine again. Every process that helps delay this recombination can enhance the photocatalytic activity of the nanocomposite. For instance, the transfer of e^-^ from the conduction band of TiO_2_ to the surface of a metallic Ag nanoparticle (Schottky junction) or PVA molecule can be the main factor for the observed enhancement in the photocatalytic behavior of the nanocomposite. It is widely believed that AgNPs can enhance photocatalytic activities via their enhanced surface plasmon resonance in the near-filed semiconductor nanostructure^45–48^. PVA aids in capturing holes through an oxidation process, effectively separating photogenerated electrons and holes^49^, an essential process for delaying the e^−^–h^+^ recombination. The generated e^−^ and h^+^ can participate in generating reactive oxygen species (ROS) such as ·O_2_^−^ and ·OH, which can participate in removing heavy metal ions through the oxidation reactions [Eqs. (9)–(16)].

9 $$ \mathop {TiO}\nolimits_{2} + \mathop {hv}\nolimits_{{}} \to \mathop {TiO}\nolimits_{2} \left( {\mathop e\nolimits_{CB}^{ - } + \mathop h\nolimits_{VB}^{ + } } \right) $$

10 $$ \mathop h\nolimits_{VB}^{ + } + \mathop H\nolimits_{2} \mathop O\nolimits_{{}} \to \mathop H\nolimits^{ + } + \left[ {\mathop {OH}\nolimits^{{\mathop \bullet \nolimits^{{}} }} } \right] $$

11 $${{\text{h}}}_{{\text{VB}}}^{+}+{\text{PVA}} \to {{\text{H}}}^{+}+\mathrm{oxidized PVA }\left(\mathrm{hole removal}\right)$$

12 $$ \mathop h\nolimits_{VB}^{ + } + \mathop {OH}\nolimits^{ - } \to \left[ {\mathop {OH}\nolimits^{{\mathop \bullet \nolimits^{{}} }} } \right] $$

13 $$ \mathop e\nolimits_{CB}^{ - } + \mathop O\nolimits_{2} \to \left[ {\mathop O\nolimits_{2}^{{\mathop \bullet \nolimits^{ - } }} } \right] $$

14 $$ \mathop {2O}\nolimits_{2}^{{\mathop \bullet \nolimits^{ - } }} + \mathop {2H}\nolimits_{2} \mathop O\nolimits_{{}} \to \mathop 2\nolimits_{{}} \left[ {\mathop {OH}\nolimits^{{\mathop \bullet \nolimits^{{}} }} } \right] + \mathop {2OH}\nolimits^{ - } + \mathop O\nolimits_{2} $$

15 $$ \mathop {ne}\nolimits_{{}}^{ - } + M^{n + } \to M $$

16 $$ M^{n + } + 2\left[ {\mathop {OH}\nolimits^{{\mathop \bullet \nolimits^{{}} }} } \right] \to MO_{n} + nH^{ + } $$

## Conclusion

In summary, a ternary Ag/TiO_2_/PVA nanocomposite has been developed, and the synthesis process was systematically studied using the experimental design to reach maximum efficiency for heavy metal ions removal. According to the DRS measurements, it was inferred that after the preparation of the nanocomposite, the bandgap energy of the TiO_2_ nanostructures was decreased to 2.9 eV, which is more efficient for light-derived photocatalytic reactions. Optimization of the pertinent factor (pH, reaction time, and photocatalyst dosage) helped to reach the maximum efficiency of 94%, 85%, 77%, and 77% for the removal of Cr^6+^, Cu^2+^, Zn^2+^, and Ni^2+^, respectively. The recyclability test showed that the photocatalyst was stable even after five successive runs. The kinetics measurements showed that the pseudo-second-order linear kinetic model fitted appropriately (regression values more than 95%) with the experimental results. In addition, the Langmuir isotherm described the adsorption of the metal ions on the photocatalyst. The observed enhanced photocatalytic activity for the heavy metal ions removal was attributed to the role of silver plasmonic nanostructures and PVA in delaying the electron–hole recombination. The synthesized Ag/TiO_2_/PVA nano-photocatalyst showed promising performance for the elimination of heavy metal ions and can be used for environmental remediation purposes.

### Supplementary Information

Supplementary Information.

## Data Availability

All data generated or analyzed during this study are included in this published article [and its supplementary information files].
