# Supplementary material for: UV-light-responsive Ag/TiO2/PVA nanocomposite for photocatalytic degradation of Cr, Ni, Zn, and Cu heavy metal ions
Source: Sci Rep. 2024 Mar 2;14:5195. doi: 10.1038/s41598-024-56059-5 (PMC11319828; doi:10.1038/s41598-024-56059-5)
Supplement: Supplementary file 1 — Supplementary Information. [file 41598_2024_56059_MOESM1_ESM.docx]

***Supplementary Information***

***Visible-light-responsive Ag/TiO_2_/PVA nanocomposite for photocatalytic degradation of Cr, Ni, Zn, and Cu heavy metal ions***

**Table S1. The designed experimental runs using the RSM for the synthesis of Ag/TiO_2_/PVA nanocomposite.**

| First day | | | |
| --- | --- | --- | --- |
| Run | **A (Time(min))** | **B (pH)** | **C (Catalyst dose) (**$\frac{\boldsymbol{g}}{\boldsymbol{L}}\boldsymbol{)}$**)** |
| 1 | **300** | **4** | **0.05** |
| 2 | **210** | **5.5** | **0.11** |
| 3 | **300** | **7** | **0.16** |
| 4 | **120** | **4** | **0.05** |
| 5 | **300** | **7** | **0.11** |
| 6 | **300** | **7** | **0.05** |
| 7 | **120** | **7** | **0.05** |
| 8 | **120** | **4** | **0.16** |
| 9 | **300** | **4** | **0.16** |
| 10 | **120** | **7** | **0.16** |
| 11 | **210** | **5.5** | **0.11** |
| 12 | **210** | **5.5** | **0.11** |
| Second day | | | |
| 13 | **210** | **5.5** | **0.11** |
| 14 | **360** | **5.5** | **0.11** |
| 15 | **210** | **8** | **0.11** |
| 16 | **210** | **5.5** | **0.2** |
| 17 | **210** | **5.5** | **0.02** |
| 18 | **60** | **5.5** | **0.11** |
| 19 | **210** | **5.5** | **0.11** |
| 20 | **210** | **3** | **0.11** |

**Table S2. The results of the BET analysis for the synthesized Ag/TiO_2_/PVA nanocomposite.**

| 5.3531 [cm^3^(STP) g^-1^] | V_m_ |
| --- | --- |
| 23.299 [m^2^ g^-1^] | **a_s,BET_** |
| 35.717 | **C** |
| 0.2733 [cm^3^ g^-1^] | **Total pore volume(*p*/*p*_0_=0.978)** |
| 46/917 [nm] | **Average pore diameter** |

**Table S3. The results of the designed experimental runs for the optimization of the metal ions removal.**

| Ni  Removal  (%) | Zn Removal  (%) | Cu Removal  (%) | Cr Removal  (%) | Catalyst dose  (g/L) | pH | Irradiation Time (min) | Run |
| --- | --- | --- | --- | --- | --- | --- | --- |
| 68.75 | 69.15 | 73.88 | 76.76 | 0.05 | 4 | 300 | 1 |
| 78.94 | 77.73 | 85.5 | 95.17 | 0.11 | 5.5 | 210 | 2 |
| 71.27 | 72.57 | 75.85 | 89.05 | 0.16 | 7 | 300 | 3 |
| 67.6 | 67.33 | 71.05 | 74.86 | 0.05 | 4 | 120 | 4 |
| 77.5 | 76.64 | 85.61 | 96.25 | 0.11 | 7 | 300 | 5 |
| 69.05 | 65.19 | 73.9 | 85.75 | 0.05 | 7 | 300 | 6 |
| 67.44 | 64.08 | 71.47 | 82.43 | 0.05 | 7 | 120 | 7 |
| 71.22 | 72.96 | 75.15 | 78.12 | 0.16 | 4 | 120 | 8 |
| 74.14 | 78.72 | 78.33 | 85.18 | 0.16 | 4 | 300 | 9 |
| 68.73 | 68.01 | 73.98 | 84.55 | 0.16 | 7 | 120 | 10 |
| 78.49 | 78.5 | 85.45 | 95.02 | 0.11 | 5.5 | 210 | 11 |
| 77.36 | 78.85 | 85.72 | 96.54 | 0.11 | 5.5 | 210 | 12 |
| 78.12 | 77.63 | 85.5 | 95.49 | 0.11 | 5.5 | 210 | 13 |
| 74.03 | 75.79 | 80.25 | 90.04 | 0.11 | 5.5 | 360 | 14 |
| 59.19 | 60.4 | 63.75 | 79.41 | 0.11 | 8 | 210 | 15 |
| 79.98 | 80.5 | 87.75 | 98.19 | 0.2 | 5.5 | 210 | 16 |
| 69.79 | 70.49 | 75.98 | 86.16 | 0.02 | 5.5 | 210 | 17 |
| 68.41 | 70 | 74.2 | 78.93 | 0.11 | 5.5 | 60 | 18 |
| 76.73 | 78.22 | 81.29 | 92.95 | 0.11 | 5.5 | 210 | 19 |
| 65.47 | 66.96 | 70.39 | 69.45 | 0.11 | 3 | 210 | 20 |

**Table S4. The ANOVA table for the removal of Cr^6+^ ions.**

|  | p-value | F-value | Mean Square | df | Sum of Squares | Source |
| --- | --- | --- | --- | --- | --- | --- |
|  |  |  | 3.491E-06 | 1 | 3.491E-06 | **Block** |
| significant | < 0.0001 | 71.12 | 0.0005 | 9 | 0.0045 | **Model** |
|  | < 0.0001 | 45.69 | 0.0003 | 1 | 0.0003 | **A-Time** |
|  | < 0.0001 | 77.42 | 0.0005 | 1 | 0.0005 | **B-pH** |
|  | < 0.0001 | 47.10 | 0.0003 | 1 | 0.0003 | **C-Catalyst dose** |
|  | 0.6933 | 0.1659 | 1.160E-06 | 1 | 1.160E-06 | **AB** |
|  | 0.1578 | 2.37 | 0.0000 | 1 | 0.0000 | **AC** |
|  | 0.1195 | 2.96 | 0.0000 | 1 | 0.0000 | **BC** |
|  | < 0.0001 | 100.44 | 0.0007 | 1 | 0.0007 | **A²** |
|  | < 0.0001 | 399.20 | 0.0028 | 1 | 0.0028 | **B²** |
|  | 0.0128 | 9.57 | 0.0001 | 1 | 0.0001 | **C²** |
|  |  |  | 6.994E-06 | 9 | 0.0001 | **Residual** |
| not significant | 0.1652 | 2.86 | 9.838E-06 | 5 | 0.0000 | **Lack of Fit** |
|  |  |  | 3.439E-06 | 4 | 0.0000 | **Pure Error** |
|  |  |  |  | 19 | 0.0045 | **Cor Total** |
|  |  | 0.9861 | R² |  |  |  |
| 0.0005 | PRESS | 0.9723 | Adjusted R² |  | 0.0026 | **Std. Dev.** |
|  |  | 0.8973 | Predicted R² |  | 1.56 | **Mean** |
|  |  | 26.5814 | Adeq Precision |  | 0.1694 | **C.V. %** |

**Table S5. The ANOVA table for the removal of Cu^2+^ ions.**

|  | p-value | F-value | Mean Square | df | Sum of Squares | Source |
| --- | --- | --- | --- | --- | --- | --- |
|  |  |  | 1.74 | 1 | 1.74 | **Block** |
| significant | < 0.0001 | 21.32 | 87.74 | 9 | 789.67 | **Model** |
|  | 0.0231 | 7.47 | 30.73 | 1 | 30.73 | **A-Time** |
|  | 0.0874 | 3.68 | 15.14 | 1 | 15.14 | **B-pH** |
|  | 0.0018 | 19.15 | 78.80 | 1 | 78.80 | **C-Catalyst dose** |
|  | 0.7725 | 0.0888 | 0.3655 | 1 | 0.3655 | **AB** |
|  | 0.9716 | 0.0013 | 0.0055 | 1 | 0.0055 | **AC** |
|  | 0.4940 | 0.5081 | 2.09 | 1 | 2.09 | **BC** |
|  | 0.0005 | 27.83 | 114.53 | 1 | 114.53 | **A²** |
|  | < 0.0001 | 143.80 | 591.77 | 1 | 591.77 | **B²** |
|  | 0.0547 | 4.87 | 20.04 | 1 | 20.04 | **C²** |
|  |  |  | 4.12 | 9 | 37.04 | **Residual** |
| not significant | 0.1949 | 2.53 | 5.63 | 5 | 28.13 | **Lack of Fit** |
|  |  |  | 2.23 | 4 | 8.91 | **Pure Error** |
|  |  |  |  | 19 | 828.45 | **Cor Total** |
|  |  | 0.9552 | R² |  |  |  |
| 264.20 | PRESS | 0.9104 | Adjusted R² |  | 2.03 | **Std. Dev.** |
|  |  | 0.6804 | Predicted R² |  | 77.75 | **Mean** |
|  |  | 13.6960 | Adeq Precision |  | 2.61 | **C.V. %** |

**Table S6. The ANOVA table for the removal of Zn^2+^ ions.**

|  | p-value | F-value | Mean Square | df | Sum of Squares | Source |
| --- | --- | --- | --- | --- | --- | --- |
|  |  |  | 0.0022 | 1 | 0.0022 | Block |
| significant | < 0.0001 | 161.69 | 73.26 | 9 | 659.33 | **Model** |
|  | < 0.0001 | 85.40 | 38.69 | 1 | 38.69 | A-Time |
|  | < 0.0001 | 139.15 | 63.04 | 1 | 63.04 | B-pH |
|  | < 0.0001 | 303.63 | 137.57 | 1 | 137.57 | C-Catalyst dose |
|  | 0.3420 | 1.01 | 0.4560 | 1 | 0.4560 | AB |
|  | 0.0037 | 15.07 | 6.83 | 1 | 6.83 | AC |
|  | 0.0714 | 4.17 | 1.89 | 1 | 1.89 | BC |
|  | < 0.0001 | 113.60 | 51.47 | 1 | 51.47 | A² |
|  | < 0.0001 | 842.48 | 381.71 | 1 | 381.71 | B² |
|  | 0.0004 | 29.99 | 13.59 | 1 | 13.59 | C² |
|  |  |  | 0.4531 | 9 | 4.08 | **Residual** |
| not significant | 0.9084 | 0.2698 | 0.2057 | 5 | 1.03 | Lack of Fit |
|  |  |  | 0.7624 | 4 | 3.05 | Pure Error |
|  | | | | 19 | 663.41 | **Cor Total** |
|  | | 0.9939 | R² |  | | |
| 14.11 | PRESS | 0.9877 | Adjusted R² |  | 0.6731 | Std. Dev. |
|  | | 0.9787 | Predicted R² |  | 72.49 | Mean |
|  |  | 41.5995 | Adeq Precision |  | 0.9286 | C.V. % |

**Table S7. The ANOVA table for the removal of Ni^2+^ ions.**

|  | p-value | F-value | Mean Square | df | Sum of Squares | Source |
| --- | --- | --- | --- | --- | --- | --- |
|  |  |  | 5.56 | 1 | 5.56 | **Block** |
| significant | < 0.0001 | 34.28 | 61.69 | 9 | 555.24 | **Model** |
|  | 0.0061 | 12.70 | 22.87 | 1 | 22.87 | **A-Time** |
|  | 0.0111 | 10.13 | 18.24 | 1 | 18.24 | **B-pH** |
|  | 0.0002 | 35.78 | 64.40 | 1 | 64.40 | **C-Catalyst dose** |
|  | 0.9836 | 0.0004 | 0.0008 | 1 | 0.0008 | **AB** |
|  | 0.4948 | 0.5063 | 0.9112 | 1 | 0.9112 | **AC** |
|  | 0.1811 | 2.10 | 3.78 | 1 | 3.78 | **BC** |
|  | 0.0002 | 37.49 | 67.47 | 1 | 67.47 | **A²** |
|  | < 0.0001 | 225.39 | 405.68 | 1 | 405.68 | **B²** |
|  | 0.0363 | 6.04 | 10.87 | 1 | 10.87 | **C²** |
|  |  |  | 1.80 | 9 | 16.20 | **Residual** |
| not significant | 0.1039 | 3.95 | 2.69 | 5 | 13.47 | **Lack of Fit** |
|  |  |  | 0.6821 | 4 | 2.73 | **Pure Error** |
|  | | | | 19 | 663.41 | **Cor Total** |
|  | | 0.9717 | R² |  | | |
| 145.62 | PRESS | 0.9433 | Adjusted R² |  | 1.34 | **Std. Dev.** |
|  | | 0.7452 | Predicted R² |  | 72.11 | **Mean** |
|  |  | 18.2429 | Adeq Precision |  | 1.86 | **C.V. %** |

**Table S8. The measured kinetics parameters for the pseudo first-order and second-order kinetic models.**

| Pseudo second-order kinetic model | | | Pseudo first-order kinetic model | | |  |
| --- | --- | --- | --- | --- | --- | --- |
| R^2^ | K_2_ × 10^3^  (mg g^-1^min^-1^) | q_e, cal_  (mg g^-1^) | R^2^ | K_1_ × 10^-4^  (min^-1^) | q_e, cal_  (mg g^-1^) |  |
| 0.985 | **0.121** | **144.509** | **0.717** | **-1.394** | **83.631** | **Cr** |
| 0.965 | **0.610** | **20.820** | **0.635** | **-6.951** | **132.778** | **Ni** |
| 0.984 | **0.720** | **31.889** | **0.518** | **-1.015** | **124.453** | **Cu** |
| 0.993 | **0.178** | **71.839** | **0.835** | **-3.111** | **116.049** | **Zn** |





**Figure S1. The efficiency of the synthesized Ag/TiO_2_/PVA nanocomposite for the photocatalytic removal of Cu^2+^ ions for five consecutive runs.**
